# Supplementary figures and images for: Mesophyll specific expression of a bacterial mercury transporter-based vacuolar sequestration machinery sufficiently enhances mercury tolerance of Arabidopsis
Source: Front Plant Sci. 2022 Aug 12;13:986600. doi: 10.3389/fpls.2022.986600 (PMC9412105; doi:10.3389/fpls.2022.986600)

**
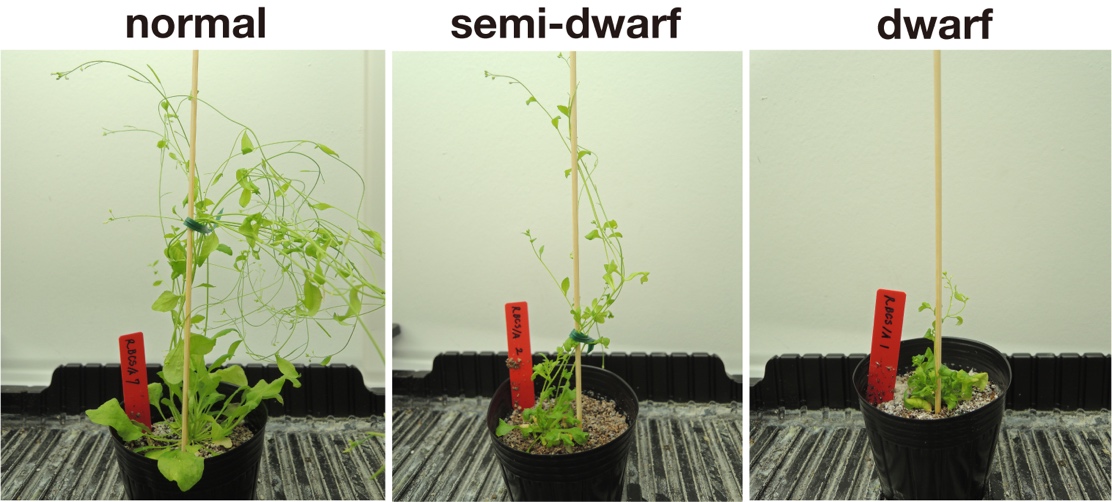
**

**Supplementary Figure S1.** Typical phenotypes of pRBCS1A-TCV transgenic T1 plants..

Supplement: Supplementary file 2 [file Data_Sheet_1.docx]
